# CAS Array: design and assessment of a genotyping array for Chinese biobanking

**DOI:** 10.1093/pcmedi/pbad002

**Published:** 2023-02-23

**Authors:** Zijian Tian, Fei Chen, Jing Wang, Benrui Wu, Jian Shao, Ziqing Liu, Li Zheng, You Wang, Tao Xu, Kaixin Zhou

**Affiliations:** National Laboratory of Biomacromolecules, Institute of Biophysics Chinese Academy of Sciences, Beijing 100101, China; College of Life Sciences, University of the Chinese Academy of Sciences, Beijing 10140, China; College of Life Sciences, University of the Chinese Academy of Sciences, Beijing 10140, China; College of Life Sciences, University of the Chinese Academy of Sciences, Beijing 10140, China; National Laboratory of Biomacromolecules, Institute of Biophysics Chinese Academy of Sciences, Beijing 100101, China; College of Life Sciences, University of the Chinese Academy of Sciences, Beijing 10140, China; Department of Mathematics and Interdisciplinary, Guangzhou Laboratory, Guangzhou 510005, China; College of Life Sciences, University of the Chinese Academy of Sciences, Beijing 10140, China; National Laboratory of Biomacromolecules, Institute of Biophysics Chinese Academy of Sciences, Beijing 100101, China; National Laboratory of Biomacromolecules, Institute of Biophysics Chinese Academy of Sciences, Beijing 100101, China; National Laboratory of Biomacromolecules, Institute of Biophysics Chinese Academy of Sciences, Beijing 100101, China; College of Life Sciences, University of the Chinese Academy of Sciences, Beijing 10140, China; College of Public Health, Guangzhou Medical University, Guangzhou 510006, China

**Keywords:** genotyping, single-nucleotide polymorphism (SNP), mitochondrial copy number, chronic disease, precision medicine, SNP array

## Abstract

**Background:**

Chronic diseases are becoming a critical challenge to the aging Chinese population. Biobanks with extensive genomic and environmental data offer opportunities to elucidate the complex gene–environment interactions underlying their aetiology. Genome-wide genotyping array remains an efficient approach for large-scale genomic data collection. However, most commercial arrays have reduced performance for biobanking in the Chinese population.

**Materials and methods:**

Deep whole-genome sequencing data from 2 641 Chinese individuals were used as a reference to develop the CAS array, a custom-designed genotyping array for precision medicine. Evaluation of the array was performed by comparing data from 384 individuals assayed both by the array and whole-genome sequencing. Validation of its mitochondrial copy number estimating capacity was conducted by examining its association with established covariates among 10 162 Chinese elderly.

**Results:**

The CAS Array adopts the proven Axiom technology and is restricted to 652 429 single-nucleotide polymorphism (SNP) markers. Its call rate of 99.79% and concordance rate of 99.89% are both higher than for commercial arrays. Its imputation-based genome coverage reached 98.3% for common SNPs and 63.0% for low-frequency SNPs, both comparable to commercial arrays with larger SNP capacity. After validating its mitochondrial copy number estimates, we developed a publicly available software tool to facilitate the array utility.

**Conclusion:**

Based on recent advances in genomic science, we designed and implemented a high-throughput and low-cost genotyping array. It is more cost-effective than commercial arrays for large-scale Chinese biobanking.

## Introduction

Chronic diseases are the major cause of mortality in the elderly.^1,2^ With the rapid progress of population aging, chronic diseases are becoming a critical public health issue and economic burden in China.^3,4^ Due to the complex gene–environment interplay in their aetiology, better understanding of the chronic disease mechanism and discovery of novel biomarkers are urgently required to facilitate precision medicine.^5,6^

Large prospective cohorts such as the UK Biobank, which collected extensive environmental information coupled with genomic data, have been proved capable of dissecting the complex aetiology of common chronic diseases.^5–8^ However, both the genetic background and environmental factors affecting those complex diseases can vary between populations.^6^ Therefore, large perspective cohort studies coupled with biobanks are essential to meet the challenge of Chinese population-specific precision medicine for the aging population.

High-throughput and cost-effective genomic techniques have advanced dramatically. Whole-genome sequencing (WGS) can identify genetic variations accurately with any allele frequency across the whole genome.^9^ While the cost of WGS has dropped significantly, single-nucleotide polymorphism (SNP) genotyping arrays remain the most cost-effective way of collecting genomic data on a biobank scale. SNP arrays focus on more informative variants among the genome to achieve higher throughput at a lower cost. Together with imputation methods, SNP arrays can generate a relatively accurate genotype, except for extremely rare variants.^9^ Imputed genotypes derived from SNP arrays can provide similar statistical power to those from WGS for genome-wide association studies (GWAS).^10,11^ However, most commercial SNP arrays were designed to maximize genome coverage and imputation accuracy in populations of European ancestry. These arrays include a significant proportion of SNPs that are monomorphic while genotyping samples from the other ethnic groups, resulting in a loss of valid information content. That is why most national biobanks worldwide have chosen to design a customized SNP array for genomic data collection.^12–14^

As large-scale biobanks of Chinese cohorts are currently underway, an SNP array optimized for large Chinese prospective cohort studies is urgently needed. The existing SNP arrays designed for the Chinese population were mostly based on small global genome reference panels such as the 1000 Genomes Project (1kGP) or the HapMap established more than a decade ago.^15,16^ With the recent advance in large-scale population sequencing in the Chinese population,^17^ genomic mapping with higher resolution offered an opportunity to design a more efficient SNP array for Chinese biobanks.

Another recent advance in human genetics is the confirmation of mitochondrial DNA copy number (MCN) as a novel biomarker of aging-related diseases and all-cause mortality.^18,19^ Studying MCN in large cohorts and biobanks was made possible by the development of methodologies that could estimate MCN through analysing raw genotyping intensity data from existing SNP arrays.^20–22^ However, none of the existing SNP arrays were optimized for MCN estimation, and had either insufficient markers or unbalanced intensities.^20,22^ Therefore, future SNP arrays could be designed to include more mitochondrial markers to facilitate MCN estimation as an extra type of genetic biomarker content for studies of ageing-related outcomes.

Here we describe the design and assessment of a genome-wide SNP array, the CAS Array, specifically optimized for cost-effective whole genome genotyping in the Chinese population. The array design took advantage of a large high-quality Chinese genomic reference panel and incorporated the latest methodological developments for MCN estimation, providing an efficient tool for precision medicine in Chinese individuals.

## Materials and methods

### Datasets

Three main datasets were used for the development and assessment of the CAS Array. The development dataset is part of the NyuWa reference panel, which includes deep (30x) WGS data of 2 641 Chinese individuals across China.^17^ It was mainly used to construct two reference panels for SNP selection and imputation validation. The evaluation dataset consists of another 384 Chinese individuals with both WGS and CAS Array data available.^23^ This was used for evaluating the genotyping accuracy and imputation performance. The validation dataset came from a large population cohort, which includes 10 162 elderlies recruited from Kunshan City, Jiangsu, China. These individuals were genotyped with the CAS Array to validate the MCN estimates by assessing their association with established age-related biomarkers recorded in the electronic health records.

### Construction of the Chinese reference panels

For array design, a tagging reference marker panel was constructed from the development dataset of 2 641 Chinese individuals with WGS variant calls. Quality control [Variant Quality Score Recalibration (VQSR) passed, SNPs only, missing rate < 0.05, minor allele count ≥ 3, quality value ≥ 30, read depth (DP) ≥ 3, and Hardy–Weinberg equilibrium (HWE), *P* value > 10^−6^] was conducted by VCFtools.^24^ A total of 17.3 M SNPs, including 5 M common (minor allele frequency (MAF) ≥ 0.05) SNPs and 71 k rare (0.001 < MAF < 0.05) coding SNPs passed the quality control and were used for GWAS tagging marker selection.

To derive the reference panel for imputation, slightly different quality control steps were applied to the development dataset. Among the SNPs passing VQSR, those with missing rate > 0.05, HWE *P* value < 10^−6^ or minor allele count < 3 were excluded. Samples that were probably contaminated (deviate ± 3 SD from mean heterozygosity rate), relatives within the third degree or abnormally recorded data were excluded. The sex of each individual was inferred by F coefficient and SNP observation on the Y chromosome. A putative XO type sample was marked as male to match the haploid state of the X chromosome. The relationship inference was done by KING software and other quality control steps were done by PLINK.^25,26^ The genotype was phased and converted to IMPUTE2 reference panel format by SHAPEIT2 software with a 0.5 Mb window size as recommended for WGS data.^27^ The genetic maps used for phasing were obtained from SHAPEIT4.^28^ The final reference panel contains 2 562 samples with 17.9 M SNPs.

### Array design

As for genotyping arrays chosen by most national biobanks, the CAS Array utilized a ThermoFisher Axiom custom array harboring up to 675 k markers. The SNP markers were selected according to three priorities. Firstly, to achieve adequate coverage of common variants for imputation-based GWAS, common SNPs on the Axiom APMRA with proven technical efficacy were anchored.^29^ They were then complemented by greedy tagging on our reference panel to cover all the common (MAF > 0.05) SNPs. The second priority was to directly type as many coding variants with MAF > 0.001 as possible in our reference panel that Axiom technical efficacy allowed. Finally, a total of 776 mitochondrial markers were selected to enable more accurate MCN estimation. Additional markers were added to the array for a wider range of applications in medical research. Markers in the human leukocyte antigen (HLA) region, pharmacokinetic variants in drug absorption, distribution, metabolism, and excretion (ADME), ancestry informative markers (AIMS), and mitochondrial markers were selected based on the reference set validated by Illumina and Affymatrix. HLA markers, ADME markers and AIMS with MAF > 0.01 in our development dataset were included while all available mitochondrial markers were included on the array.

### Evaluation of coding variants coverage

Coding variants were more likely to be identified as clinically relevant.^30^ However, clinical translation of such knowledge of precision medicine requires high genotyping accuracy to maintain reasonable sensitivity and specificity, which could be better achieved by directly genotyping rather than using imputed genotypes. The coverage of coding variants with MAF > 0.001 was examined on the latest ChinaMAP reference panel.^31^ Variants position, alleles labels, and frequencies derived from WGS data of 10 588 Chinese individuals were downloaded and annotated with ANNOVAR.^32^ There were 107.4 k variants marked as coding variants with MAF > 0.001 in ChinaMAP. The coding variants coverage of the CAS Array was defined as the proportion of variants having matched position and alleles with the designed markers on the arrays relative to the total of 107.4 k variants on ChinaMAP.

### Evaluation of genotyping accuracy

Genotyping accuracy of the CAS Array was evaluated by calculating the concordance rate between WGS calls and array genotyping results in the array evaluation dataset. Quality control of WGS data was the same as that applied to the imputation reference panel. Array genotyping SNPs were called by APT software following the manufacturer's instructions.^33^ Five samples having inconsistent sex or that were duplicated were removed by PLINK.^26^ The array genotyping call rate was defined as the proportion of recommended variants relative to the total number of designed markers on the array. Within these successfully called SNPs on the array, concordance rate was calculated as the proportion of concordant genotypes relative to all non-missing variant calls from WGS.

### Evaluation of imputation performance

The evaluation dataset was also used to evaluate the imputation performance of the CAS Array as compared to eight commonly used commercial arrays, including Genome-Wide Human SNP Array 6.0 (Affy SNP6), Axiom Precision Medicine Research Array (Axiom PMRA), Axiom Asia Precision Medicine Research Array (Axiom APMRA), Infinium Global Screening Array (Illumina GSA), Infinium Asian Screening Array (Illumina ASA), Infinium HumanOmni1 (Illumina Omni1), Infinium OmniExpress (Illumina OE), and Infinium OmniZhongHua (Illumina OZH). Manifest files were downloaded from the respective official websites of these arrays and the positions of the markers were converted to genome build hg38 by UCSC liftOver.^34^ Genotypes with matching physical position and alleles were extracted from the WGS dataset as simulated genotyping calls. Low-quality variants including those with call rate < 0.95, MAF < 0.01, or HWE *P* value < 10^−6^ were excluded before imputation. Autosomes and chromosome X genotypes of each array were phased by SHAPEIT2 using the genetic map from SHAPEIT4.^27,28^ The reference strands were aligned to our Chinese reference panel derived from the NyuWa reference panel by Genotype Harmonizer.^35^ Imputation was performed by IMPUTE2 with the same reference panel.^36^

The imputation performance of each array was evaluated by comparing the imputed genotypes with the original WGS outputs. We used imputation *r*^2^, discordance rate, and imputation-based genomic coverage to assess the performance of the arrays as in previous studies.^13,14,37^ The imputation *r*^2^ was defined as the squared Pearson correlation *r*^2^ between the allele dosages of WGS and imputed genotypes. The discordance rate was defined as the proportion of the mismatching genotypes between WGS results and the most possible genotypes at each site generated by imputation. Coverage was defined as the proportion of the variants having imputation *r*^2^ greater than a given threshold (typically *r*^2^ > 0.8). Average imputation *r*^2^ and discordance rate was calculated for each array. Coverage of common SNPs (MAF ≥ 0.05) and low-frequency SNPs (0.01 ≤ MAF < 0.05) were calculated separately for the arrays.

### MCN estimation

MCN estimation was conducted in a similar manner as implemented by two previous MCN estimation pipelines, MitoPipeline and AutoMitoC.^20,21^ In brief, the MCN was estimated by the intensity of fluorescent signal of mitochondrial markers indicating the segments of mitochondrial DNA captured by the corresponding probes. The intensities of autosomal markers were used as a reference to capture latent confounding factors such as batch effects and variation in DNA concentrations. Firstly, raw genotyping intensity files were processed for quality control by APT Software.^33^ Genotype calls and normalized signal intensity were also generated by APT. Log R ratios (LRRs) were calculated as an intensity measure and corrected for GC content to adjust for genomic waves by PennCNV.^38,39^ To select high-quality markers for MCN estimation, PLINK and BLAST+ were used for quality control.^26,40^ Markers with multiple alignment of percentage of identical matches > 80% were excluded for potential off-target. For autosomal markers, additional quality control including call rate > 95%, HWE *P*-value > 10^−6^, linkage disequilibrium (LD)-pruning (*r*^2^ < 0.3), and maximum spacing was done. After filtering, 47 102 autosomal markers and 166 mitochondrial markers were left as high-quality markers for MCN estimation. Principal component analysis (PCA) was applied on the LRRs of high-quality autosomal markers generating 80 PCs using R.^41^ The LRRs of high-quality mitochondrial markers were adjusted by regressing out the PCs of the autosomal markers. The final MCN estimates were extracted from the adjusted mitochondrial LRRs by PCA and converted to a standard normal distribution. After excluding samples with low genotyping quality (call rate < 0.95), fluctuating LRR (LRR SD > 0.35), inconsistent sex calling, or without available phenotype data, the validation data set was finally used to examine the association between estimated MCN and age-related biochemical traits such as white blood cells count (WBC), haemoglobin (HEMO), and platelets (PLT). The same pipeline was also applied on the evaluation dataset, where MCN estimated from array data could be compared directly with MCN estimated from WGS as twice the ratio of the sequencing depth between mitochondrial reads and autosomal reads.

## Results

### Content of CAS Array

We designed an Axiom SNP array based on the large Chinese NyuWa genome reference panel of 2641 individuals.^17^ The CAS Array includes a total of 652 429 SNPs selected for different purposes (Table 1). Of these, 525 k variants were selected as genome-wide tagging SNPs (MAF > 0.01) for GWAS. Another 108 k of the markers offer high direct coverage of coding variants with MAF > 0.001 in the Chinese population. In addition to the small numbers of SNPs selected for other types of precision medicine investigations, 776 mitochondrial SNP markers were included for MCN estimation.

**Table 1.** Summary of the contents of CAS Array.

Category	Number of markers	Proportion of markers
GWAS tagging markers	525 113	80.49%
Coding variants	108 261	16.59%
HLA markers	14 843	2.28%
ADME markers	1 403	0.22%
AIMS	2 033	0.31%
Mitochondrial markers	776	0.12%
Total	652 429	100.00%

### Genotyping call rate and accuracy

Call rate and accuracy of the CAS Array were evaluated by assaying 384 Chinese individuals with both the CAS Array and WGS. Of the 652 577 SNP markers on the array (including technical markers of Axiom), 645 327 were genotyped and passed quality control, resulting in a raw call rate of 98.89%. Of the 582 342 non-ambiguous variants that overlapped between CAS Array and WGS, the average concordance rate across samples was 99.89%. These results indicate that the in-house genotyping accuracy of the CAS Array was comparable to most commercial SNP arrays.^13,14,42^

### Coverage of coding variants in the Chinese population

To evaluate the coverage of coding variants in the Chinese population, we utilized the large external genome reference panel of the ChinaMAP.^31^ Out of the 107 403 coding variants with MAF > 0.001 in the ChinaMAP, 74 470 (69.3%) were directly captured by the CAS Array and passed quality control. Compared to other commonly used commercial SNP arrays, CAS Array has a much higher direct coverage of coding variants that are more relevant to precision medicine (Table 2).

**Table 2.** Direct coverage of coding variants (MAF > 0.001) in ChinaMAP WGS results of CAS Array and commonly used commercial SNP arrays.

Array name	Number of coding variants covered	Proportion of coding variants covered
CAS Array	74 470	69.3%
Affy SNP6	6 528	6.1%
Axiom PMRA	6 917	6.4%
Axiom APMRA	31 155	29.0%
Illumina GSA	12 732	11.9%
Illumina ASA	22 657	21.1%
Illumina Omni1	27 584	25.7%
Illumina OE	16 740	15.6%
Illumina OZH	22 463	20.9%

### Imputation performance

**Figure 1.**
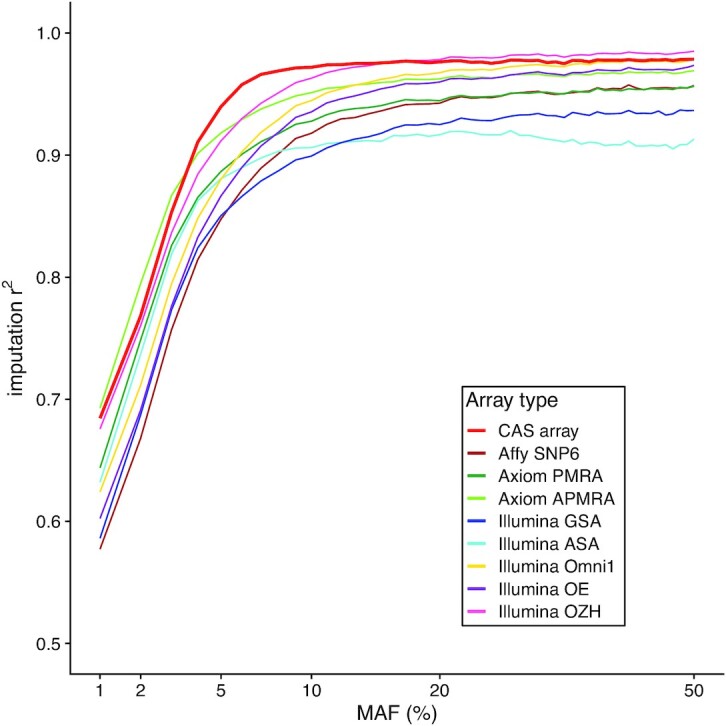
Comparison of imputation *r*^2^ between CAS Array and other SNP arrays. Simulated genotyping results of CAS Array and eight commonly used commercial SNP arrays were extracted from whole-genome sequencing genotypes of 384 Chinese individuals. Imputation was conducted with the simulated array genotyping results and the accuracy was evaluated by imputation *r*^2^ stratified by minor allele frequency.

Imputation performance was evaluated on both accuracy and coverage using the evaluation dataset. Within the post quality control WGS data, there are 4.2 M common SNPs (MAF ≥ 0.05) and 1.6 M low-frequency SNPs (0.01 ≤ MAF < 0.05). Figure 1 shows the imputation *r*^2^ distribution across the allele frequency spectrum for the nine arrays. CAS Array demonstrated the highest overall imputation accuracy, probably due to the fact that up to 90.6% of its limited contents are common and informative to imputation. A similar pattern was observed when discordance rate was used to evaluate accuracy (supplementary Tables 1 and 2, see online supplementary material). When imputed genotypes with *r*^2^ > 0.8 were set as the good coverage target, CAS Array achieved rates of 98.3% and 63.0% for common and rare SNPs respectively, higher than most commercial arrays containing more SNP markers. These results indicate that CAS Array outperformed most commonly used commercial SNP arrays on imputation accuracy and genome coverage despite its limited SNP content.

### MCN estimation and validation

**Figure 2.**
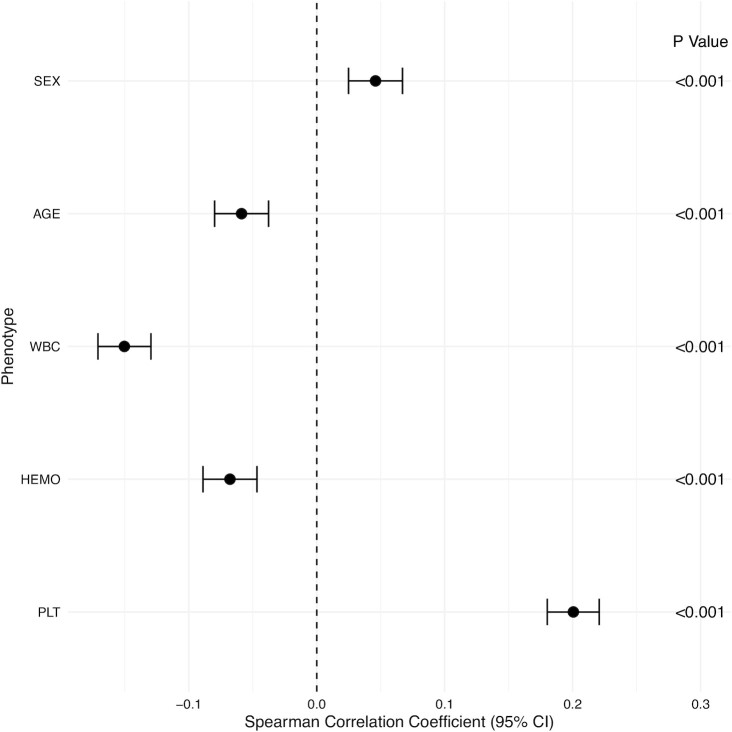
Association between mitochondrial copy number estimates and different phenotypes. The plot shows the spearman rank correlation coefficients with 95% confidence intervals (CI) and *P*-values of the association between mitochondrial copy number estimated from CAS Array and corresponding phenotypes in 8 584 Chinese individuals.

We developed a pipeline to estimate the MCN from raw genotyping intensity data of the CAS Array and applied it to the validation dataset. After quality control, 378 individuals in the evaluation dataset had their MCN estimated by 47 878 high-quality markers, including 134 mitochondrial markers. The MCN estimated from CAS Array was positively correlated with the MCN estimated from WGS (spearman correlation rho = 0.52, *P* < 2.2 × 10^−16^). For the validation dataset, a total of 8 584 individuals passed quality control and their MCN was estimated by 47 268 high-quality markers, including 166 mitochondrial markers. As shown in Fig. 2, MCN estimates were significantly associated with age, sex, WBC, HEMO, and PLT, in keeping with previous studies.^22,43,44^ The pipeline was packed into an R package available on GitHub (https://github.com/Zijian-Tian/CASMCN).

## Discussion

We designed an Axiom SNP array that is suitable for high-throughput and low-cost genotyping in large Chinese cohorts. With a limited content of ∼675 k markers, the CAS Array achieved a relatively high genotyping accuracy and high genome coverage via imputation. Given the design features of direct coverage on coding variants and MCN estimation, the CAS Array should become a good choice for biobank-scale genotyping and precision medicine in Chinese population.

As with other custom-designed genotyping arrays for biobanks, the main purpose of CAS Array is to facilitate cost-effective large-scale GWAS via imputation.^36^ The comparison of post-imputation accuracy and genome coverage shows that CAS Array is generally more suitable than most commercial arrays for GWAS in the Chinese population. Axiom APMRA achieved better performance than the CAS array at the low-frequency (MAF < 0.05) end of SNP distribution, but only at the cost of ∼150 k extra rare markers on the array (supplementary Table 3, see online supplementary material). At the high-frequency (MAF > 0.2) end, the Illumina OmniZhongHua (OZH) array outperformed CAS Array at the cost of genotyping a total of 1.1 M SNPs with reduced throughput. Therefore, on the balance of cost effectiveness, CAS Array is a more reliable and attractive option for low-cost and high-throughput genotyping in the Chinese population.

In addition to facilitating the marker selection on the CAS array, the high-quality Chinese reference panel also played an important role in improving its imputation performance. Our results show that all SNP arrays had better imputation performance when using the large NyuWa Chinese reference panel compared to the widely-used 1kGP reference panel,^17,45^ especially on low-frequency SNPs (Fig. 1, supplementary Figs. 1–5, see online supplementary material). This advantage is likely driven by the fact that our reference panel was not only larger than the extended 1kGP panel but also more representative of the Chinese population. As described in the original publications, the 1kGP reference panel included 585 east Asian individuals and only 163 of them are southern Han Chinese.^45^ In contrast, the NyuWa reference panel consists of 2 562 Chinese individuals from 23 of 34 administrative divisions in China.^17^ Therefore, the CAS Array would serve genotyping of Chinese individuals better, especially with the large Chinese imputation reference panels that are increasingly available.

The designing priority to directly genotype more coding variants is another key feature of CAS Array. This group of variants has been proven by accumulating GWAS results to be the most likely type of causal variants for a wide range of complex phenotypes.^46^ The direct calling of these variants would enable more accurate genotyping than imputation. In turn, the downstream association analyses and genetic risk profiling would be more powerful and accurate with these directly assayed genotypes. More importantly, these more accurate genotype calls would also benefit the translation of genomic knowledge into potential clinical practice. As suggested by multiple biobanks around the world, pre-emptive genotyping of key pharmacogenetic variants, which are mostly coding variants, would benefit from more reliable genotype data to achieve high specificity.^47^

CAS Array is the first genotyping array designed with MCN estimation in mind, aiming to better serve the investigations into complex age-related diseases. Compared to other commonly used arrays with dozens to 300 mitochondrial probes, CAS Array harbors 776 mitochondrial SNP markers. Therefore, it has more comprehensive data and statistical power to estimate MCN. We also implemented an array-specific pipeline to estimate MCN from raw genotyping intensity signals. Using the large validation dataset, we further demonstrated that the MCN estimated from CAS Array was indeed associated with established biomarkers, paving the path to use the array for more precision medicine research in the elderly.

The CAS Array design is inherently limited by the total number of markers it can carry, in order to meet the requirement of cost-effective genotyping. However, with the support of more comprehensive Chinese reference genome panels, the CAS Array outperformed most commercial arrays in terms of imputation-based GWAS for complex trait gene mapping. Although coding variants were prioritized on the CAS Array, higher coverage of variants with translational potential is still limited. A more purpose-built translation-oriented genotyping array will become a useful tool when more Chinese-specific functional variants are discovered by large-scale biobank studies. It is also worth noting that the accuracy of array-based MCN estimation is prone to technical fluctuations, and is thus more appropriate for large sample investigations.

In conclusion, we designed and implemented the CAS Array based on a large comprehensive Chinese reference genome panel. Albeit restricted by the SNP content, its relatively high genotyping accuracy and imputation performance, high coverage of coding variants, and convenient MCN estimation, together make the array a cost-effective tool for large Chinese biobanking and precision medicine studies.

## Supplementary Material

pbad002_Supplemental_Tables_and_Figures
