# Supplementary material for: CAS Array: design and assessment of a genotyping array for Chinese biobanking
Source: Precis Clin Med. 2023 Feb 23;6(1):pbad002. doi: 10.1093/pcmedi/pbad002 (PMC10031742; doi:10.1093/pcmedi/pbad002)
Supplement: pbad002_Supplemental_Tables_and_Figures [file pbad002_supplemental_tables_and_figures.docx]

# Supplementary Materials

**Supplementary Table 1. Comparison of average imputation r^2^ between CAS Array and other SNP arrays.**

| **Array type** | **MAF ≥ 1%** | **MAF ≥ 5%** | **1% ≤ MAF < 5%** |
| --- | --- | --- | --- |
| CAS array | 92.5% | 97.4% | 79.5% |
| Affy SNP6 | 86.9% | 93.5% | 69.5% |
| Axiom PMRA | 89.2% | 94.1% | 76.2% |
| Axiom APMRA | 91.7% | 95.9% | 80.5% |
| Illumina GSA | 86.1% | 91.8% | 70.8% |
| Illumina ASA | 86.7% | 91.0% | 75.3% |
| Illumina Omni1 | 89.8% | 96.0% | 73.6% |
| Illumina OE | 88.7% | 95.2% | 71.6% |
| Illumina OZH | 92.0% | 97.2% | 78.1% |

MAF, minor allele frequency; SNP, single nucleotide polymorphism; Affy SNP6, Affymetrix Genome-Wide Human SNP Array 6.0; Axiom PMRA, Axiom Precision Medicine Research Array; Axiom APMRA, Axiom Asia Precision Medicine Research Array; Illumina GSA, Infinium Global Screening Array v3.0; Illumina ASA, Infinium Asian Screening Array v1.0; Illumina Omni1, Infinium HumanOmni1; Illumina OE, Infinium OmniExpress; Illumina OZH, Infinium OmniZhongHua.

**Supplementary Table 2. Comparison of average discordance rate between CAS Array and other SNP arrays.**

| **Array type** | **MAF ≥ 1%** | **MAF ≥ 5%** | **1% ≤ MAF < 5%** |
| --- | --- | --- | --- |
| CAS array | 0.97% | 0.95% | 1.04% |
| Affy SNP6 | 2.06% | 2.21% | 1.67% |
| Axiom PMRA | 1.87% | 2.10% | 1.25% |
| Axiom APMRA | 1.34% | 1.47% | 1.00% |
| Illumina GSA | 2.56% | 2.93% | 1.57% |
| Illumina ASA | 2.91% | 3.51% | 1.30% |
| Illumina Omni1 | 1.33% | 1.29% | 1.41% |
| Illumina OE | 1.56% | 1.58% | 1.53% |
| Illumina OZH | 0.96% | 0.89% | 1.15% |

MAF, minor allele frequency; SNP, single nucleotide polymorphism; Affy SNP6, Affymetrix Genome-Wide Human SNP Array 6.0; Axiom PMRA, Axiom Precision Medicine Research Array; Axiom APMRA, Axiom Asia Precision Medicine Research Array; Illumina GSA, Infinium Global Screening Array v3.0; Illumina ASA, Infinium Asian Screening Array v1.0; Illumina Omni1, Infinium HumanOmni1; Illumina OE, Infinium OmniExpress; Illumina OZH, Infinium OmniZhongHua.

**Supplementary Table 3. Number of simulated genotyping SNPs of SNP arrays after each step of extraction.**

| **Array Name** | **Original SNP count** | **Available  SNP count** | **Informative  SNP count** |
| --- | --- | --- | --- |
| CAS Array | 652,573 | 638,332 (97.8%) | 591,306 (90.6%) |
| Affy SNP6 | 929,577 | 807,943 (86.9%) | 700,497 (75.4%) |
| Axiom PMRA | 902,152 | 542,503 (60.1%) | 424,892 (47.1%) |
| Axiom APMRA | 799,049 | 647,703 (81.1%) | 521,138 (65.2%) |
| Illumina GSA | 650,712 | 479,663 (73.7%) | 368,767 (56.7%) |
| Illumina ASA | 652,118 | 555,752 (85.2%) | 486,317 (74.6%) |
| Illumina Omni1 | 1,044,726 | 854,864 (81.8%) | 751,480 (71.9%) |
| Illumina OE | 713,212 | 646,163 (90.6%) | 584,097 (81.9%) |
| Illumina OZH | 1,169,167 | 963,750 (82.4%) | 869,699 (74.4%) |

Original SNPs were defined as SNPs retained after lifted the manifest file of the SNP arrays to genome build hg38 and were the reference set when computing the percentage in the table. Available SNPs were defined as the original SNPs found in the WGS result. Informative SNPs were defined as the available SNPs which passed the quality control process (call rate ≥ 95%, MAF ≥ 0.01 and HWE p value ≥ 10^-6^). SNP, single nucleotide polymorphism; Affy SNP6, Affymetrix Genome-Wide Human SNP Array 6.0; Axiom PMRA, Axiom Precision Medicine Research Array; Axiom APMRA, Axiom Asia Precision Medicine Research Array; Illumina GSA, Infinium Global Screening Array v3.0; Illumina ASA, Infinium Asian Screening Array v1.0; Illumina Omni1, Infinium HumanOmni1; Illumina OE, Infinium OmniExpress; Illumina OZH, Infinium OmniZhongHua


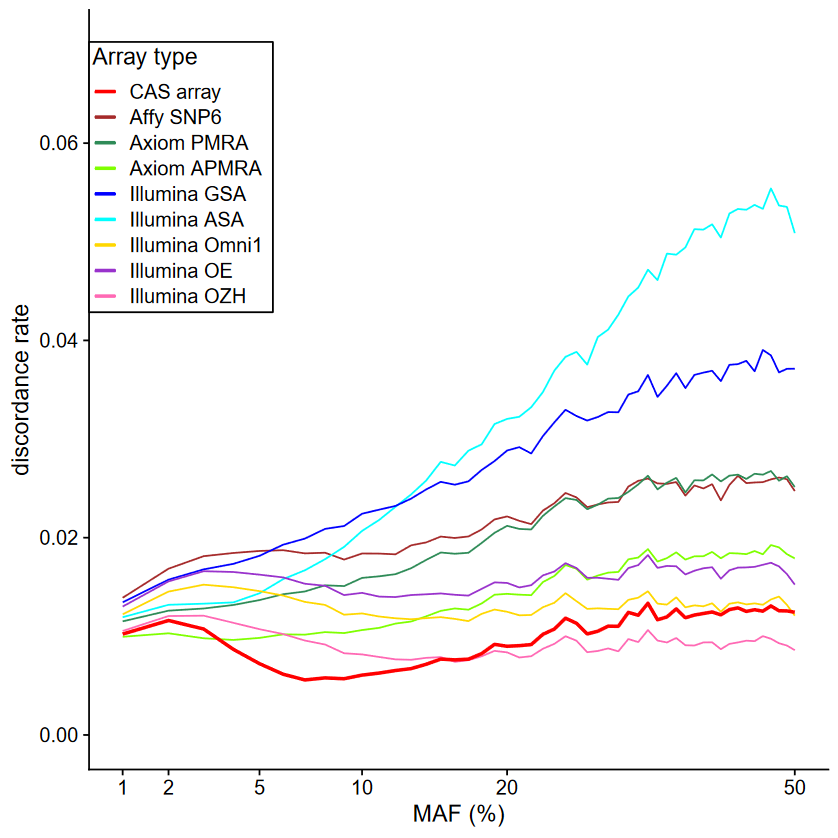


**Supplementary Figure 1. Comparison of discordance rate between CAS Array and other SNP arrays.** Simulated genotyping results of CAS Array and 8 commonly used commercial SNP arrays were extracted from whole-genome sequencing genotype of 384 Chinese individuals. Imputation was conducted with the simulated array genotyping results and the accuracy was evaluated by discordance rate of the SNPs stratified by minor allele frequency. SNP, single nucleotide polymorphism; Affy SNP6, Affymetrix Genome-Wide Human SNP Array 6.0; Axiom PMRA, Axiom Precision Medicine Research Array; Axiom APMRA, Axiom Asia Precision Medicine Research Array; Illumina GSA, Infinium Global Screening Array v3.0; Illumina ASA, Infinium Asian Screening Array v1.0; Illumina Omni1, Infinium HumanOmni1; Illumina OE, Infinium OmniExpress; Illumina OZH, Infinium OmniZhongHua.


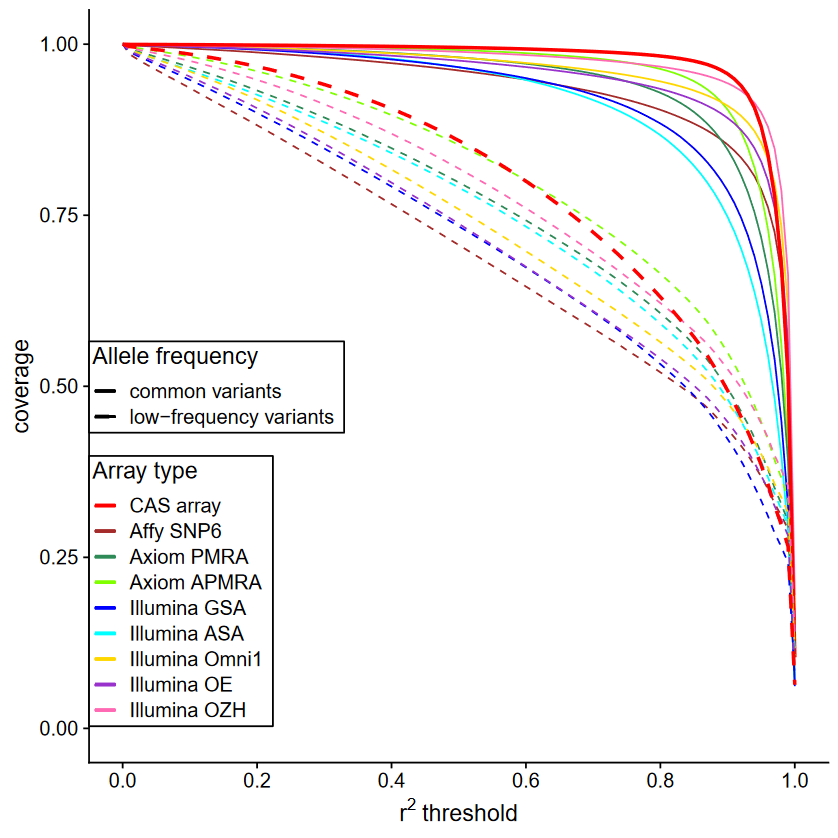


**Supplementary Figure 2. Comparison of imputation-based genomic coverage between CAS Array and other SNP arrays.** Genomic coverage was defined as the proportion of SNPs with imputation r^2^ greater than the given threshold over the intersection of the SNPs between the imputation reference panel and whole-genome sequencing result. Common SNPs (MAF ≥ 5%) and low-frequency SNPs (1% ≤ MAF < 5%) were plotted separately. MAF, minor allele frequency; SNP, single nucleotide polymorphism; Affy SNP6, Affymetrix Genome-Wide Human SNP Array 6.0; Axiom PMRA, Axiom Precision Medicine Research Array; Axiom APMRA, Axiom Asia Precision Medicine Research Array; Illumina GSA, Infinium Global Screening Array v3.0; Illumina ASA, Infinium Asian Screening Array v1.0; Illumina Omni1, Infinium HumanOmni1; Illumina OE, Infinium OmniExpress; Illumina OZH, Infinium OmniZhongHua.


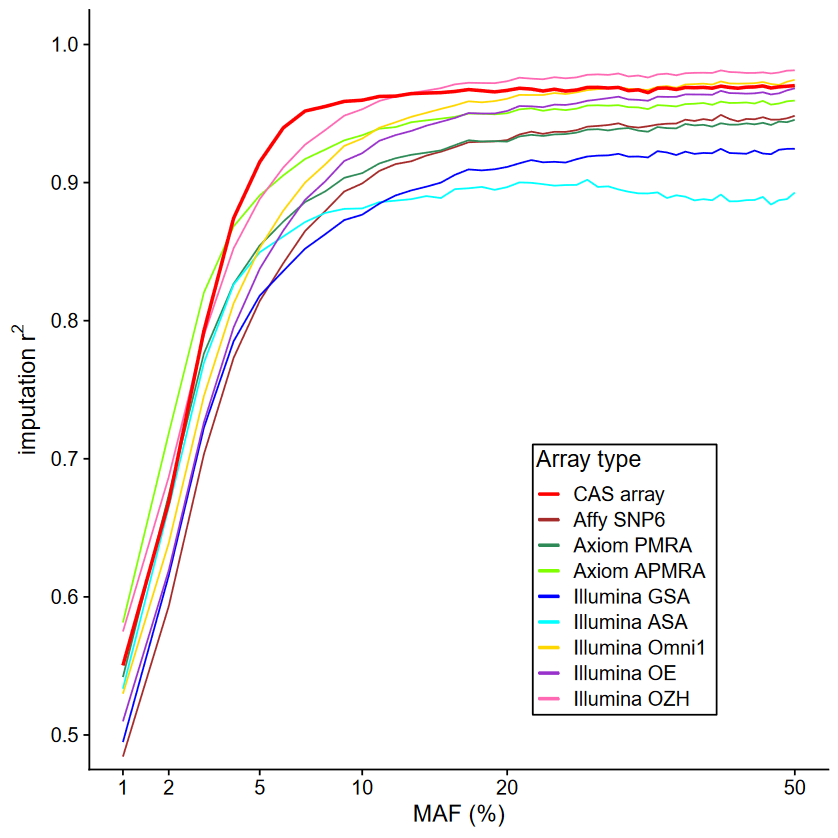


**Supplementary Figure 3. Comparison of imputation r^2^ between CAS Array and other SNP arrays with 1000 Genomes Project imputation reference panel.** Simulated genotyping results of CAS Array and 8 commonly used commercial SNP arrays were extracted from whole-genome sequencing genotype of 384 Chinese individuals. Imputation was conducted with the simulated array genotyping results and reference panel from the expanded 1000 Genomes Project whole-genome sequencing genotypes. The accuracy was evaluated by imputation r^2^ stratified by minor allele frequency. SNP, single nucleotide polymorphism; Affy SNP6, Affymetrix Genome-Wide Human SNP Array 6.0; Axiom PMRA, Axiom Precision Medicine Research Array; Axiom APMRA, Axiom Asia Precision Medicine Research Array; Illumina GSA, Infinium Global Screening Array v3.0; Illumina ASA, Infinium Asian Screening Array v1.0; Illumina Omni1, Infinium HumanOmni1; Illumina OE, Infinium OmniExpress; Illumina OZH, Infinium OmniZhongHua.


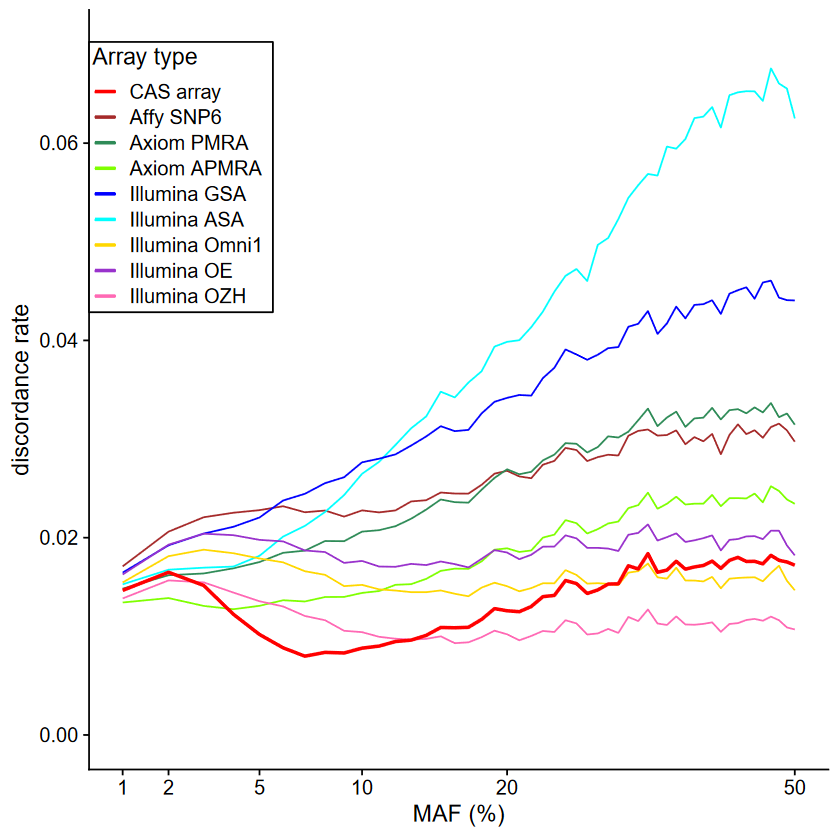


**Supplementary Figure 4. Comparison of discordance rate between CAS Array and other SNP arrays with 1000 Genomes Project imputation reference panel.** Simulated genotyping results of CAS Array and 8 commonly used commercial SNP arrays were extracted from whole-genome sequencing genotype of 384 Chinese individuals. Imputation was conducted with the simulated array genotyping results and reference panel from the expanded 1000 Genomes Project whole-genome sequencing genotypes. The accuracy was evaluated by discordance rate of the SNPs stratified by minor allele frequency. SNP, single nucleotide polymorphism; Affy SNP6, Affymetrix Genome-Wide Human SNP Array 6.0; Axiom PMRA, Axiom Precision Medicine Research Array; Axiom APMRA, Axiom Asia Precision Medicine Research Array; Illumina GSA, Infinium Global Screening Array v3.0; Illumina ASA, Infinium Asian Screening Array v1.0; Illumina Omni1, Infinium HumanOmni1; Illumina OE, Infinium OmniExpress; Illumina OZH, Infinium OmniZhongHua.


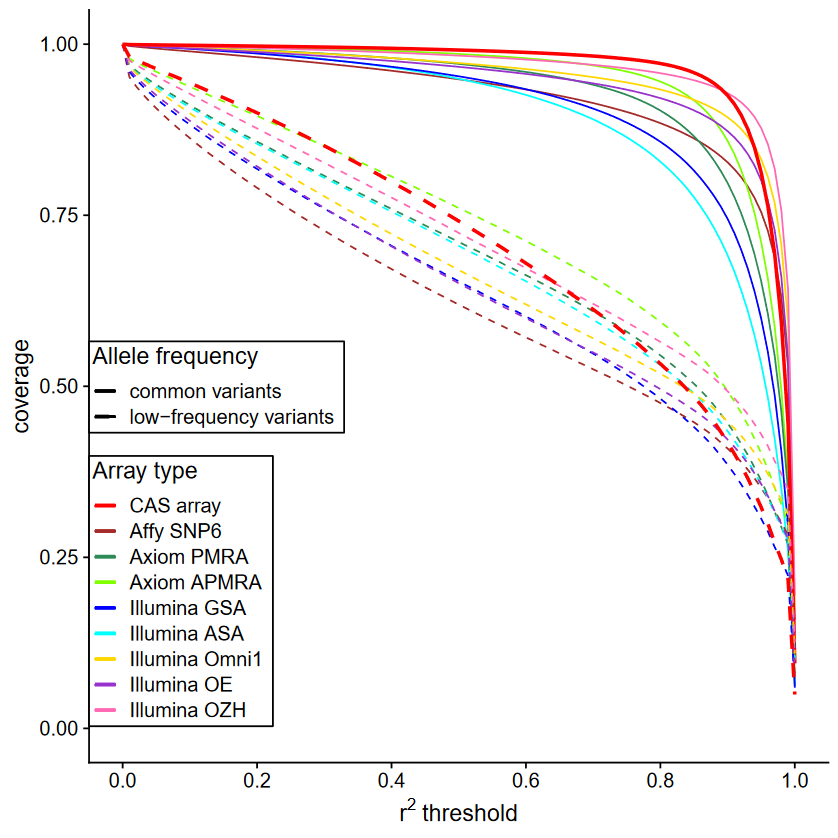


**Supplementary Figure 5. Comparison of imputation-based genomic coverage between CAS Array and other SNP arrays with 1000 Genomes Project imputation reference panel.** Genomic coverage was defined as the proportion of SNPs with imputation r^2^ greater than the given threshold over the intersection of the SNPs between the imputation reference panel and whole-genome sequencing result. Common SNPs (MAF ≥ 5%) and low-frequency SNPs (1% ≤ MAF < 5%) were plotted separately. MAF, minor allele frequency; SNP, single nucleotide polymorphism; Affy SNP6, Affymetrix Genome-Wide Human SNP Array 6.0; Axiom PMRA, Axiom Precision Medicine Research Array; Axiom APMRA, Axiom Asia Precision Medicine Research Array; Illumina GSA, Infinium Global Screening Array v3.0; Illumina ASA, Infinium Asian Screening Array v1.0; Illumina Omni1, Infinium HumanOmni1; Illumina OE, Infinium OmniExpress; Illumina OZH, Infinium OmniZhongHua.
